# Ascorbate Depletion: A Critical Step in Nickel Carcinogenesis?

**DOI:** 10.1289/ehp.7605

**Published:** 2005-02-10

**Authors:** Konstantin Salnikow, Kazimierz S. Kasprzak

**Affiliations:** Laboratory of Comparative Carcinogenesis, National Cancer Institute at Frederick, National Institutes of Health, Frederick, Maryland, USA

**Keywords:** ascorbate, carcinogenesis, collagens, extracellular matrix, hypoxia-inducible transcription factor, metals, nickel, protein hydroxylation

## Abstract

Nickel compounds are known to cause respiratory cancer in humans and induce tumors in experimental animals. The underlying molecular mechanisms may involve genotoxic effects; however, the data from different research groups are not easy to reconcile. Here, we challenge the common premise that direct genotoxic effects are central to nickel carcinogenesis and probably to that of other metals. Instead, we propose that it is formation of metal complexes with proteins and other molecules that changes cellular homeostasis and provides conditions for selection of cells with transformed phenotype. This is concordant with the major requirement for nickel carcinogenicity, which is prolonged action on the target tissue. If DNA is not the main nickel target, is there another unique molecule that can be attacked with carcinogenic consequences? Our recent observations indicate that ascorbate may be such a molecule. Nickel depletes intracellular ascorbate, which leads to the inhibition of cellular hydroxylases, manifested by the loss of hypoxia-inducible factor (HIF)-1α and - 2α hydroxylation and hypoxia-like stress. Proline hydroxylation is crucial for collagen and extracellular matrix assembly as well as for assembly of other protein molecules that have collagen-like domains, including surfactants and complement. Thus, the depletion of ascorbate by chronic exposure to nickel could be deleterious for lung cells and may lead to lung cancer.

The high consumption of metal-containing products by industrial societies inevitably leads to environmental pollution by heavy metals, including nickel(II), chromium(VI), and cobalt(II), at all stages of production, use, and disposal. Human occupational exposure to metals occurs primarily via inhalation and ingestion of metal-containing dusts and is particularly high among stainless steel welders, miners, and metallurgy workers. The environmental exposure mostly results from burning of fossil fuels. Accumulation of metals in the human body can cause different diseases, including cancer. There is sufficient evidence in humans for the carcinogenicity of Cr(VI) and Ni(II) [International Agency for Research on Cancer (IARC) 1990], which are not known to be essential in humans, and limited experimental animal data support Co(II) carcinogenicity [Bucher et al. 1999; National Toxicology Program (NTP) 1998].

How can metals be carcinogenic? One possible answer is based on the ability of metals to generate reactive oxygen species and other reactive intermediates (Kasprzak 2002) or react directly with DNA (Kasprzak 2002; Kasprzak et al. 2003; O’Brien et al. 2003). Indeed, several transition metals can generate reactive oxygen species in biologic fluids at physiologic pH, but this does not explain why the relatively weak redox-active Ni(II) is as good a carcinogen as the oxidatively very active Cr(VI). Moreover, Cr(VI) is a better carcinogen than Cr(III), and yet Cr(III) is much better in DNA binding reactions than nickel or Cr(VI) (O’Brien et al. 2003). In addition, the highly redox-active metals iron and copper, which also bind to DNA more avidly than does Ni(II) (Kasprzak et al. 1986; Trotta et al. 2002), are only weakly carcinogenic, if at all (Desoize 2003; Theophanides and Anastassopoulou 2002).

Another explanation may be based on the known ability of carcinogenic metals to facilitate DNA damage through inhibition of DNA repair enzymes (Hartwig et al. 2002) or binding to histones (Bal et al. 2000). In both cases, it is assumed that the ultimate target of free radicals or metals is DNA and that the mechanisms of carcinogenesis must include genotoxic effects. However, metals such as Ni(II) are only weakly genotoxic/mutagenic (Biggart and Costa 1986; Fletcher et al. 1994; Mayer et al. 1998). In addition, one major requirement for Ni(II) carcinogenicity is prolonged action on the target tissue (Kasprzak et al. 2003), which is typical of tumor promoters acting through epigenetic mechanisms, rather than tumor initiators, which are mutagenic. Therefore, Ni(II) compounds with limited solubility and long retention in biologic fluids are stronger carcinogens than are easily soluble compounds (Kasprzak et al. 2003). So, considering the above, it is probably time to investigate how metals can promote carcinogenesis. Also, we should shift our attention from DNA to proteins and/or other molecules that offer a much broader and more vulnerable target for metal interactions. One such target is l-ascorbic acid, or vitamin C.

The notion that ascorbate is involved in resistance to neoplasms was introduced and advocated by Linus Pauling (Cameron et al. 1979). This notion was based on demonstration of low ascorbate reserves in cancer patients, and later on results of clinical trials where survival time of cancer patients was prolonged by ascorbate supplementation (Cameron and Pauling 1976, 1978). It is well known that transition metals can interact with and destroy ascorbate (Buettner and Jurkiewicz 1996). The role of ascorbate as an antioxidant molecule has been appreciated for years, but only recently ascorbate was found to play a crucial role in hydroxylation reactions that determine interactions and functioning of thousands of cellular proteins. The hydroxylation is catalyzed by a variety of nonheme iron-containing dioxygenases, including prolyl, asparaginyl, and lysyl hydroxylases (Hewitson et al. 2003; Myllyharju and Kivirikko 2004), and novel DNA repairing enzymes, human ABH2 and ABH3 proteins (Sedgwick 2004), which require α -ketoglutarate (2OG) as cofactor. Most important, iron in these enzymes is maintained in the active Fe(II) form by ascorbate (Myllyla et al. 1984; Tschank et al. 1994). Thus, the activity of these enzymes depends on two factors, iron and ascorbate. Both may be targets for Ni(II) and other carcinogenic metals. Can this lead to carcinogenesis? Here, we present some of the evidence suggesting this possibility. We discuss one possible way—there are certainly others.

## “Oxygen Sensor” Senses Nickel

In rats, intrarenal injection of nickel subsulfide resulted first in a pronounced erythrocytosis and eventually in the development of local tumors (Jasmin and Solymoss 1975; Morse et al. 1977; Sunderman 1984). The high erythrocyte counts were mediated by increased erythropoietin (EPO) production. Exposure of human hepatoma cells (Hep3B or HepG2) to low oxygen atmosphere, or to increasing amounts of Ni(II) or Co(II), resulted in a dose-dependent enhancement of EPO mRNA expression (Goldberg et al. 1988). These findings led to the hypothesis that the induction of EPO by Co(II) or Ni(II) 2005. resulted from the substitution of the Fe(II) ion in an “oxygen sensor” (Goldberg et al. 1988). Reduced oxygen delivery to the kidney is the physiologic stimulus to EPO production, and EPO transcription is now known to be under the control of the hypoxia-inducible factor 1 (HIF-1) (Semenza and Wang 1992). HIF-1 DNA-binding activity is similarly induced by hypoxia or Co(II) chloride treatment, as originally described in Hep3B cells (Wang and Semenza 1993). In fact, exposure of cells to Co(II) was used to induce sufficient HIF-1 α in cell nuclear extracts to allow biochemical purification (Wang and Semenza 1995).

The “oxygen sensor” hypothesis implies that Ni(II) may interfere with Fe(II) metabolism. Iron and nickel belong to the eighth group of transition metals, with atomic numbers 26 and 28, respectively. Fe(II) and Ni(II) have similar ionic radii of 0.74 Å and 0.69 Å and similar ligand affinity (Nieboer and Richardson 1980). This suggests that, in theory, Ni(II) could be accommodated at an enzymatic protein Fe(II)-binding site, and substitution is possible. However, because Ni(II) is not as redox active as Fe(II), the substitution would inactivate the enzyme. A good example is phthalate dioxygenase that can be poisoned by divalent metals. This enzyme contains two iron-binding sites. A Rieske-type [2Fe-2S] cluster serves as an electron-transferring cofactor, and a mononuclear iron site is the putative site for substrate oxygenation, similar to that predicted in prolyl hydroxylases [prolyl hydroxylase domain (PHD) enzymes] (Batie and Ballou 1990). Any of the metal ions, Fe(II), Co(II), manganese(II), Ni(II), or zinc(II), can occupy the mononuclear site, but only Fe(II) is competent for effecting hydroxylation (Batie et al. 1987).

Although the substitution of metals in the enzymes seems possible, no experimental data indicate that this actually occurs in living cells. Most existing data were obtained *in vitro* using recombinant or biochemically isolated proteins. In living cells, the concentration of free transition metals is maintained at very low levels. This must be true for both essential and nonessential metals. Because most transition metals are dangerous for cells, they exist inside the cells bound to storage proteins or are transferred to the enzymes with the help of special metallochaperones. Such a model has been developed for copper, and undoubtedly similar mechanisms exist for other essential metals (O’Halloran and Culotta 2000). Thus, at present, the hypothesis of *in situ* Fe(II) substitution by Ni(II) in the cellular “oxygen sensor” remains to be tested. However, besides the iron substitution, the poisoning of “oxygen sensor” may be explained in an alternative way when the critical role of ascorbate as an iron reductant is considered.

Depletion of intracellular ascorbate produces a phenotype observed in hypoxic cells or in cells with mutated von Hippel-Lindau (VHL) protein. Importantly, hypoxia is a common feature of neoplastic tumors (Hockel and Vaupel 2001). The molecular response to hypoxia is mediated by the HIF-1 transcription factor that stimulates angiogenesis, changes energy metabolism and iron homeostasis, and promotes cell survival in the affected tissues (Semenza 2003). Therefore, in order to survive, tumor cells should have high HIF activity. Like oxygen deprivation, exposure of cells to Co(II) or Ni(II) also results in the induction of HIF-1 and production of hypoxia-like responses (Maxwell and Salnikow 2004).

**Figure 1:**
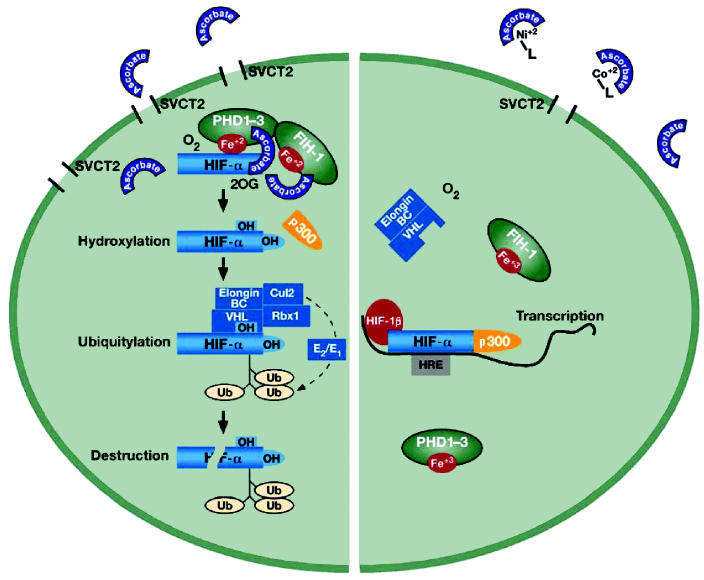
Induction of hypoxia-inducible genes in hypoxia or after metal exposure. Hydroxylation of pro-lines in HIF-α (HIF-1α and -2α ) proteins by the iron-containing hydroxylases, PHD1–3, results in interaction with VHL–ubiquitin ligase complex and proteosomal destruction (left). Hydroxylation of asparagine by FIH-1 hydroxylase prevents HIF-α binding to a transcriptional co-activator p300. The hydroxylation reaction requires oxygen as a substrate and ascorbate as an iron-reducing agent. Ascorbate is not produced in cells and delivered through sodium-dependent ascorbate transporter SVCT2. In hypoxia, HIF-α proteins cannot be hydroxylated because of low oxygen levels; in metal-exposed cells, HIF-α hydroxylation is prevented by low ascorbate levels (right). Both conditions lead to the accumulation of HIF-α and formation of HIF-1 transcription complex.

HIF-1 transcription factor is composed of one α and one β subunit (Maxwell and Salnikow 2004). The α subunit is the regulatory component of the HIF-1 complex and is unique to the hypoxic response (Figure 1). Under normoxic conditions, this protein is virtually undetectable in most cells because of hydroxylation and rapid proteasomal destruction, but it can accumulate after exposure to proteasomal inhibitors such as lactacystin or MG-132 (Salceda and Caro 1997). Accumulation of the HIF α subunit in the presence of hypoxia or Ni(II) implies that proteasomal degradation of the protein is impaired by these exposures. The β subunit of HIF-1 is constitutively expressed. In hypoxia or after metal exposure, the α subunit dimerizes with a β subunit and translocates to the nucleus. The produced HIF-1 complex binds to the HIF response element’s initiating transcription of hypoxia-inducible genes.

The β subunit is also involved in xenobiotic responses where HIF-1β forms a dimer with the aryl hydrocarbon receptor (AhR). Therefore, an alternative name for HIF-1β is ARNT (AhR nuclear translocator) (Wang and Semenza 1995). It is noteworthy that although ARNT expression itself is not directly affected by Ni(II), the expression of AhR-dependent genes can be significantly suppressed by Ni(II) exposure (Davidson et al. 2003). The down-regulation of AhR-dependent genes has an important toxicologic implication. It may lead to a decrease in toxicant removal. Because cross-talk between the AhR-dependent pathway and HIF-dependent pathway has been described, it is conceivable that Ni(II) affects a major regulatory factor upstream of both pathways. Indeed, the similarity in effects of Ni(II), hypoxia, and the inhibitor of 2OG-dependent dioxygenases (dimethyloxalylglycine) suggests that iron and 2OG-dependent dioxygenases are involved in regulation of both pathways.

## Hydroxylation-Dependent Protein–Protein Interactions

It has been shown recently that the key protein–protein interactions that govern the HIF-1 activation are regulated by oxygen through enzymatic hydroxylation of specific proline and asparagine residues. Hydroxylation of proline residues 402 and 564 in the oxygen-dependent degradation domain of HIF-1α leads to its interaction with the VHL tumor suppressor protein, a part of the ubiquitin–ligase complex (Figure 1) (Ivan et al. 2001; Jaakkola et al. 2001; Maxwell et al. 1999). This is followed by ubiquitylation and rapid proteosomal degradation of HIF-1α . The structural basis for this specific interaction is provided by the introduction of hydroxyl group at the proline 4-position that then facilitates hydrogen bonding with VHL’s Ser-111 and His-115 residues. This is sufficient to enable discrimination between non-hydroxylated and hydroxylated HIF-α (HIF-1α and -2α ) proteins (Hon et al. 2002; Ivan et al. 2001; Jaakkola et al. 2001; Masson et al. 2001; Min et al. 2002).

**Figure 2:**
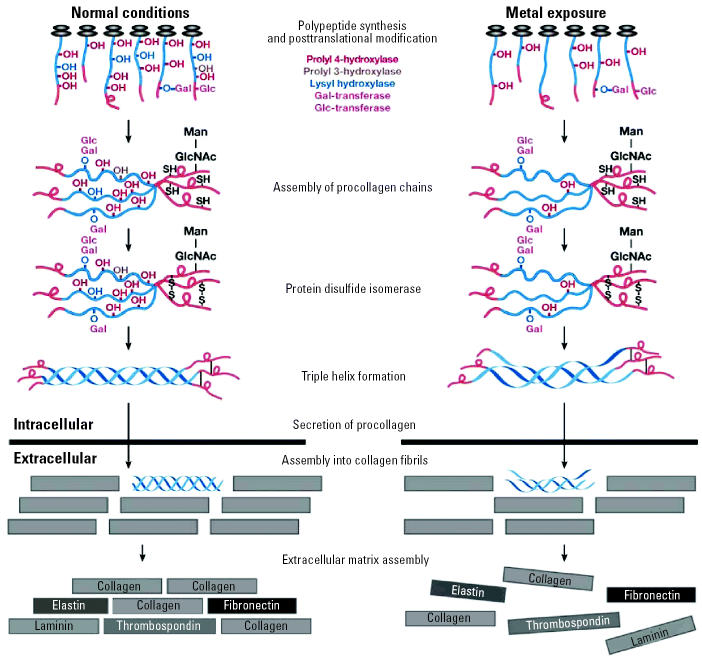
Metals can affect formation of extracellular matrix (ECM) by inhibition of collagen hydroxylation (based on Myllyharju and Kivirikko 2004). The hydroxylation of prolyl and lysyl residues in collagen peptide is catalyzed by members of the 2OG dioxygenase family (i.e., prolyl 4-hydroxylase, prolyl 3-hydroxylase, and lysyl hydroxylase). All enzymes require a nonhelical substrate and recognize XaaYaaGly amino acid sequence. The reaction mechanisms of all three hydroxylases are similar, and each requires Fe(II), 2OG, molecular O_2_, and ascorbate. The hydroxylation allows assembly of triple helices that are stable at body temperature. Secreted protocollagen is assembled into collagen fibrils, a major structural component of ECM. Depletion of intracellular ascorbate by metals leads to the inhibition of collagen hydroxylation and disorganization of ECM.

Besides proline, hydroxylation of asparagine and lysine residues was found to be important for protein–protein interactions. Thus, asparagine hydroxylation allows for complex formation between HIF-1α and the CREB-binding protein and p300 transcriptional coactivators that in this way also become involved in the transcriptional control of HIF-dependent responses (Freedman et al. 2002). The hydroxylation of proline and lysine residues in collagen molecules serves to form and stabilize the collagen triple helices under physiologic conditions (Figure 2). Collagen chains that do not contain 4-hydroxyproline cannot fold into triple helical molecules that are stable at body temperature (Pihlajaniemi et al. 1991). Ascorbate plays a key role in the trimerization step of three α -chains and/or in the subsequent triple-helix formation of collagen (Yoshikawa et al. 2001). The collagen superfamily today includes at least 27 collagen types with at least 42 distinct polypeptide chains, and > 20 additional proteins with collagen-like domains (Myllyharju and Kivirikko 2004). Altogether, they make up 30% of cellular protein mass. In addition to collagens, proline hydroxylation probably affects interaction of many more cellular proteins, because 4-hydroxyproline residues are ubiquitous in proteins with collagen-like domains (Myllyharju and Kivirikko 2004). Among such proteins are lung surfactant proteins A and D, also known as collectins, or collagen-like domain and calcium-dependent lectin domain proteins (Crouch and Wright 2001). Surfactant proteins A and D are found primarily in the alveolar fluid in lungs of mammalians. Their primary structure is highly conserved among different mammalian species. Similarly to the loss of hydroxylation in collagen, the failure of surfactant protein hydroxylation results in a decrease in melting temperature of 9°C and failure of assembly of the collagen-like domain (Garcia-Verdugo et al. 2003).

In addition to surfactants A and D, C1q protein, the first component of complement, also known as defense collagen because of its involvement in the immunomodulation of inflammatory and allergic responses of the lung, contains 4-hydroxyproline in its collagen domain (Lu et al. 2002). Therefore, the loss of hydroxylation of C1q protein should affect inflammatory responses in the lung.

At the tissue level, the loss of hydroxyprolines in the collagen-like domains of surfactants A and D could impair their functions. Surfactant inactivation and deficiencies have, indeed, been associated with a variety of lung diseases, including pneumonia, asthma, and acute respiratory distress syndrome (Griese 1999). A similar pattern of lung diseases has been reported for workers occupationally exposed to Ni(II) (Kelleher et al. 2000). Numerous data indicate that ascorbate deficiency may be an underlying factor in the pathophysiology of asthma and acute respiratory distress syndrome (Kelly 1998; Kongerud et al. 2003). They are in agreement with the hypothesis that Ni(II) may target ascorbate in the lungs of exposed individuals.

There are at least three functional classes of hydroxylases belonging to nonheme dioxygenases (Ryle and Hausinger 2002). Among them are two long-known collagen prolyl-4-hydroxylases (Myllyharju 2003), the more recently identified FIH-1 (factor inhibiting HIF), and PHD1–3, asparaginyl and prolyl hydroxylases, responsible for HIF-α protein hydroxylation (Epstein et al. 2001; Hewitson et al. 2002; Lando et al. 2002; Mahon et al. 2001). The third class, characterized most recently, includes alkyl DNA dioxygenases, such as the *Escherichia coli* AlkB dioxygenase and its two human homologues, ABH2 and ABH3, which facilitate a novel mechanism of DNA repair (Sedgwick 2004). In the presence of oxygen, these enzymes can specifically hydroxylate alkyl groups on 1-methyladenine and 3-methylcytosine (Duncan et al. 2002). The requirement of iron for the reaction as well as inhibition of hydroxylase activity in crude cell extracts by iron chelators suggests that these enzymes are iron dependent. Inhibition by *N*-oxalylamino acid derivatives, first developed for use with collagen prolyl 4-hydroxylases, further implies that these hydroxylases are also dependent upon 2OG as a cosubstrate (Cunliffe et al. 1992). The source of the hydroxyl oxygen is always (> 95%) molecular oxygen (Hayaishi and Nozaki 1969). In the course of the hydroxylation reaction, the enzyme-bound Fe(II) splits dioxygen into two atoms, being converted itself to Fe(III) or Fe(IV). The exact mechanism of the reaction is not known, but it is likely that after full cycle iron is returned to Fe(II). Any interference with the reaction, not clearly defined yet, will leave iron at a higher oxidation state. The role of ascorbate is to reduce iron back to Fe(II) and thus reactivate the enzyme (de Jong et al. 1982; Myllyla et al. 1984). This scenario implies that iron depletion or oxidation leads to the loss of hydroxylase enzymatic activity and, again, points to ascorbic acid as a crucial player.

## Extracellular Matrix, Ascorbate, and Tumor Promoters

The extracellular matrix (ECM) is composed of a great variety of molecules and includes collagen family, elastic fibers, glycosoaminoglycans and proteoglycans, and adhesive glycoproteins. ECM functions to bind cells together, to provide conditions for growth factor–receptor interaction, and to control growth and differentiation. It plays an important role in maintaining structural integrity and regulating cell polarity, migration, and survival. The major component of the ECM is collagen (Myllyharju and Kivirikko 2004). In cutaneous tissues (i.e., skin), type I collagen accounts for 80–90% of all collagenous proteins in ECM (Kielty et al. 1993). Defects in ECM can lead to developmental abnormalities and cancer.

Neoplastic progression is associated with specific changes in the ECM. These changes can be observed in very early stages of tumor development and involve, first of all, disorganization of the basement membrane and a decrease in the deposition of a new collagen. The exposure to tumor-promoting agents is characterized by similar changes. For example, skin treatment with the tumor-promoting phorbol ester 12-*O*-tetradecanoylphorbol-13-acetate (TPA) leads to a reduction of the collagen content of the mouse dermis (Marian 1987; Marian and Mazzucco 1985). Both collagen degradation and synthesis are affected by the carcinogens, but the effect on degradation is more pronounced (Pinto et al. 1970). Because collagen assembly greatly depends on ascorbate, the decrease in the ascorbate level during spontaneous or carcinogen-induced tumor progression could be a key factor in the ECM disorganization (Preist 1970). Indeed, it was shown that TPA decreases transport activity of both sodium-dependent vitamin C transporters SVCT1 and SVCT2 (Daruwala et al. 1999). TPA-induced depletion of intra-cellular ascorbate can be surmounted by the addition of ascorbate that is manifested by the reversion of the cell-transforming activity of TPA (Sivak and Tu 1980).

The reduced collagen expression at the early step of carcinogenesis may cause disturbed keratinocyte adhesion to the basement membrane (Parikka et al. 2003). In *in vitro* experiments, anchorage-independent growth represents a good test for malignant transformation of cells, emphasizing an important role of cell attachment and ECM in the carcinogenesis process. Indeed, the transformed phenotype of mouse fibroblasts that did not grow in semisolid medium could be completely reverted by ascorbate exposure (Benedict et al. 1982). Moreover, addition of ascorbate inhibited soft agar growth of rat osteosarcoma or human neuroblastoma cells (Girgert et al. 1999; Sugimoto et al. 1986).

## Maintenance of Ascorbate Levels

Ascorbate is a well-known antioxidant required by all mammalian cells for proper functioning (Padayatty et al. 2003). Although rodents (except for guinea pigs) produce ascorbate in the liver, humans are unable to synthesize ascorbate. This inability is likely to be reflected in the ascorbate levels in the blood serum, given normal consumption of ascorbate-containing food. The comparison of ascorbate levels in different species suggests that humans probably have lower levels than do rodents. Thus, a number of studies have provided values of 14.9–58.8 μ M ascorbate in blood serum for healthy volunteers receiving the recommended dietary allowance of 60 mg vitamin C daily (Levine et al. 1996). In another study, Dawson et al. (1999) described average levels of ascorbate of 35–40 μ M. In rats, Gumuslu et al. (2000) reported an average ascorbate level of 63 μ M in the blood serum, and in mice, Atanasova et al. (2004) and Kuo et al. (2004) found between 96 and 125 μ M ascorbate. These species-related differences in ascorbate levels should be taken into consideration when results of animal experiments are analyzed. For example, the inhalation exposure to carcinogenic nickel compounds resulted in increased incidence of alveolar/bronchiolar neoplasms and adrenal medulla neoplasms in male and female rats but not in mice (Dunnick et al. 1995); unfortunately, in this study ascorbate levels were not analyzed in blood or tissues of experimental animals. Under our concept, it is conceivable that a higher level of ascorbate in mice protected these animals from nickel-induced tumor development. Additionally, the appearance of adrenal medulla neoplasms in exposed rats can also be mediated by the effect of Ni(II) on ascorbate levels because adrenal medulla has the highest requirement for ascorbate in the body (Patak et al. 2004). Exposure to cobalt sulfate causes alveolar/bronchiolar neoplasms both in rats and in mice (Bucher et al. 1999), and cobalt sulfate is more efficient in the depletion of intra-cellular ascorbate (Salnikow et al. 2004). Could it contribute to the effectiveness of cobalt in animal carcinogenesis?

**Figure 3:**
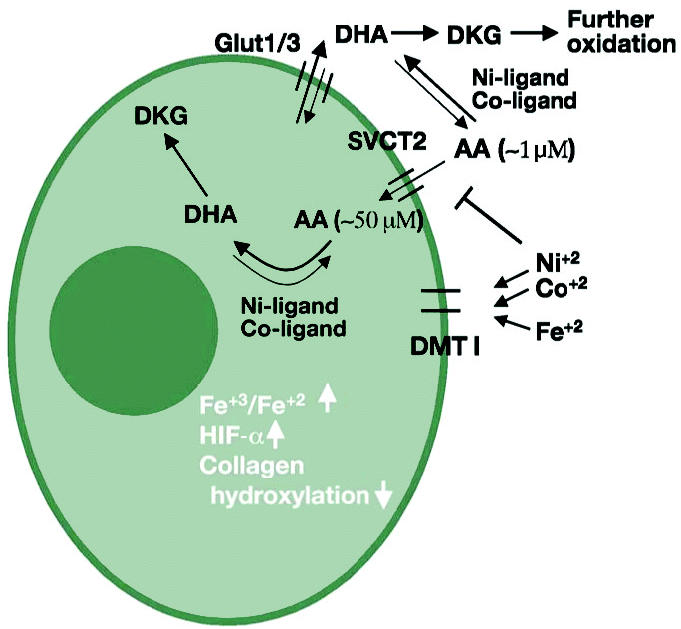
Metal-induced ascorbate depletion. Numerous iron-containing hydroxylases that are involved in collagen, carnitine, catecholamine, and tyrosine metabolism all require ascorbic acid (AA) to maintain iron in reduced form. In tissue culture media, the level of AA is 1–5 μ M, and it is dependent on the source of serum. In cells AA is accumulated > 10- to 50-fold compared with the media levels because of uptake of both DHA, via Glut 1, and AA, via sodium-dependent ascorbate transporter SVCT2. Once oxidized to DHA, ascorbate can be reduced inside the cell, or be released from cells to the media in the form of DHA. In the media, DHA can be reduced to AA and transported back to cells. Ni(II) and Co(II), after binding to ligands, are capable of catalyzing ascorbate oxidation to DHA both in the media and inside the cell. In the presence of metals, DHA is not stable and can be hydrolyzed to diketogulonate (DKG) and other products. Arrows show the preferential pathway of metabolic conversion of ascorbate. Both Ni(II) and Co(II) could also block ascorbate transport into cells. The loss of intracellular ascorbate results in accumulation of oxidized iron [increased ratio Fe(III):Fe(II)] in the enzymes, which causes their inactivation.

The maintenance of necessary intracellular ascorbate level in humans depends on dietary intake and subsequent distribution into cells. Thus, the intracellular level of ascorbate strongly depends on the ascorbate levels in the blood or in the culture media. Ascorbic acid and its oxidized form, dehydroascorbic acid (DHA), both can be transported into and out of cells (Figure 3). Glucose transporters GLUT 1 and GLUT 3 mediate transport of DHA (Vera et al. 1993), whereas ascorbic acid enters cells through sodium-dependent transporters SVCT1 and SVCT2 (Takanaga et al. 2004). Inside the cell, DHA is immediately reduced to ascorbic acid, which accumulates at higher than outside concentrations (Nualart et al. 2003). The intracellular level of ascorbate is therefore regulated by its oxidation status, uptake, and efflux. In culture medium, the only source of ascorbate is serum. Thus, we found the concentration of ascorbate in Eagle minimal essential medium supplemented with 10% serum to be between 1 and 5 μ M (Salnikow et al. 2004). At the same time, human lung epithelial cells grown in this medium accumulated up to 60 μ M ascorbate. Nonetheless, it is likely that cells in tissue culture do not receive adequate amounts of ascorbate (Quievryn et al. 2002). Therefore, the ascorbate values reported for cells *in vitro* probably are lower than those existing in cells of corresponding tissues *in vivo*. This may explain rather high *K*_m_ values reported for prolyl hydroxylases. Thus, the *K*_m_ values found for PHDs were found in a range of 140–180 μ M, whereas for collagen prolyl-4-hydroxylase *K*_m_ was 300 μ M (Hirsila et al. 2003). The activity of DNA dioxygenases ABH2 and ABH3 was also found to be ascorbate dependent; however, the *K*_m_ values for the enzymes were not measured (Duncan et al. 2002). The maintenance of intracellular level of ascorbate therefore is critical for the activity of specific hydroxylases.

## Ascorbic Acid Interactions with Transition Metals

The intra- and extracellular levels of ascorbate can be greatly affected by the interaction with metals (Figure 3). In the absence of molecular oxygen, the reactions of ascorbic acid with certain transition metal ions, especially Fe(III), Cu(II), mercury(II) [Hg(II)], or Cr(VI), result in their reduction and oxidation of ascorbic acid to DHA, for example, 2Fe(III) + ascorbic acid → 2Fe(II) + DHA + 2H^+^ (Tsao 1997). However, in the presence of oxygen or other oxidants, such as hydrogen peroxide or lipid peroxides, these metals act predominantly as catalysts in a chain of partially radical reactions (Khan and Martell 1967). The metal catalyst cycles between its original and lower valency state. These reactions include the formation of a binary complex between the metal and mono-deprotonated ascorbic acid molecule, and dioxygen coordination by the bound metal. Once ascorbate is oxidized, the metal is dissociated because it cannot be bound to DHA.

Other metals, such as Zn(II), Ni(II), Co(II), or lead(II) [Pb(II)], which do not accept electrons at neutral pH, can neither oxidize ascorbic acid by themselves nor serve as catalysts for oxidation with molecular oxygen (Fridman et al. 1979; Khan and Martell 1967). However, this is only true for simple aqueous solutions in which only binary metal–ascorbate complexes can be formed. In tissue fluids and cell culture media, the situation is radically different owing to the presence of a wide variety of natural metal complexing agents (chelators), such as amino acids, peptides, proteins, nucleotides, and glutathione, which may bind and profoundly affect the redox potential of these metals (Holmes and Williams 2000) or their dioxygen coordination capacity (Boca 1983; Van Horn et al. 2003) and thus make them catalytically active toward ascorbate oxidation. Most important, such ligands may form ternary complexes with the metal and ascorbic acid (Fridman et al. 1983). Among natural chelators, histidine and histidine-containing peptides, especially those having the N-terminal XaaYaaHis motif (e.g., albumin), play the major role in biologic binding of transition metals such as Cu(II), Ni(II), or Co(II) (Harford and Sarkar 1995). The ascorbic acid in these complexes was found to be oxidized by ambient oxygen much faster than was ascorbic acid alone (Puplikova and Savich 1969). Besides histidine, other amino acids, including glycine, alanine, serine, phenylalanine, and glutamic acid, have been found to form ternary complexes with Ni(II) and ascorbic acid, with stability of those with histidine and glutamic acid being highest (Fridman et al. 1979, 1981, 1983).

Transition metal chelation by components of biologic fluids other than ascorbic acid may also facilitate the oxidative destruction of the latter through more indirect mechanisms, for example, those involving Fenton-like chemistry with metabolic H_2_O_2_ and generation of reactive oxygen and other radical species from lipid peroxides. These are avidly scavenged by the ascorbate anion, thus lowering its tissue levels (Buettner and Jurkiewicz 1996). The best examples of this effect would include facilitation by Ni(II), a non-Fenton metal, reactivity with O_2_ or H_2_O_2_ through chelation with polyglycyl, polyalanyl, or histidine-containing oligopeptides (Bossu et al. 1978; Datta et al. 1993).

In cells, the oxidation of ascorbate to DHA is readily reversible owing to specific enzymatic systems, including DHA reductase, glutaredoxins, and protein disulfide isomerase, and depends on glutathione or other thiols as electron donors (Tsao 1997). However, unlike ascorbic acid, DHA undergoes relatively fast (*t*_1/2_ ~ 2–15 min at pH 7) spontaneous hydrolysis to a product, 2,3-diketogulonic acid, which cannot be regenerated. In addition, DHA and 2,3-diketogulonic acid may also be further oxidized by molecular oxygen, H_2_O_2_, and some metal oxyanions (permanganate, chromate) to products such as threonic acid, oxalic acid, and various smaller products (e.g., carbon dioxide) that cannot be reverted back to ascorbic acid (Deutsch 1998).

There also is a possibility that metals can form complexes with derivatives of ascorbic acid, such as ascorbate-2-sulfate, 2-*O*-β -glucuronide, and 2-*O*-α -glucoside that have been found in mammalian cells (Davey et al. 2000), and ascorbate-6-phosphate, which has been found in bacteria (Zhang et al. 2003) but has not yet been detected in mammalian cells.

## The Key Role of Ascorbate in Nickel Toxicity and Carcinogenesis

The effect of Ni(II) compounds on cells is complex and most likely involves modulation of many metabolic pathways. Recently, we found that Ni(II) exposure greatly depleted intracellular ascorbate, which results in the activation of the HIF-1 transcription factor and up-regulation of hypoxia-inducible genes (Salnikow et al. 2004). The addition of ascorbate to the media caused an increase in intra-cellular ascorbate and reversed both metal-induced stabilization of HIF-1α and HIF-1–dependent gene transcription. Recently, the important role of ascorbate in down-regulation of HIF-1α level in tumor cell lines has been demonstrated (Knowles et al. 2003).

The depletion of intracellular ascorbate, as the initial step of Ni(II) toxicity, has profound effects on cellular and tissue metabolism. It shifts cellular homeostasis toward a more transformed phenotype. Here, we are considering three major modifications in cellular metabolism due to the depletion of intra-cellular ascorbate by Ni(II): *a*) induction of hypoxia-like stress, *b*) impairment of assembly of proteins with collagen-like domains, and *c*) inactivation of ascorbate-dependent DNA repair enzymes.

Exposure of human or rodent cells to Ni(II) stabilizes HIF-1α and induces HIF-1 transcriptional activity, followed by the induction of glycolytic enzymes and glucose transporters (Salnikow et al. 1999, 2003). The up-regulation of the glycolytic pathway may be beneficial for cell survival, and if Ni(II) exposure is prolonged, it may select cells that maintain a high glycolytic rate and thereby acquire a phenotype similar to cancer cells, as described by Warburg (1956). In addition, other hypoxia-inducible genes, including BCL-2 binding protein *Nip3*, *EGLN1*, hypoxia-inducible gene 1 (*HIG1*), prolyl-4 hydroxylase, and cyclin G2, and growth factors, such as platelet-derived growth factor BB and vascular endothelial growth factor (VEGF), are up-regulated by Ni(II) in an HIF-dependent manner (Kuwabara et al. 1995; Namiki et al. 1995; Salnikow et al. 2003; Steinbrech et al. 2000).

Although the benefit of up-regulation of some hypoxia-inducible genes for cell survival and transformation is not understood, the up-regulation of others is clearly beneficial. Thus, up-regulation of cyclin G2 ceases cell proliferation, allowing conservation of energy (Horne et al. 1997). The up-regulation of growth factors has an antiapoptotic effect and helps to sustain cell growth by both autocrine and paracrine regulatory mechanisms (Tran et al. 2002). In the long term, VEGF as a major tumor angiogenesis and vascular permeability factor can support cell survival and growth via enhanced blood supply. Angiogenesis can be further stimulated by the suppression of anti-angiogenic factors such as thrombospondin I. Both Ni(II) and hypoxia suppress thrombospondin I expression (Laderoute et al. 2000; Salnikow et al. 1994). The importance of HIF activation and expression of HIF target genes for evolution of a more malignant phenotype was shown in an animal model system (Elson et al. 2000). Additionally, immunostaining studies using monoclonal antibodies against the HIF α -subunits demonstrated increased HIF-1α expression in about 53% of malignant tumors, including colon, breast, gastric, lung, ovarian, pancreatic, prostate, and renal cell carcinomas, melanomas, and glioblastomas as compared with the respective normal tissues (Zhong et al. 1998). In another study, increased protein levels of both HIF-1α and HIF-2α were detected in about 54% of tumors screened (Talks et al. 2000).

Similarly to hypoxia, Ni(II) exposure up-regulates HIF-1 and transactivates HIF-1–dependent genes that are critical for invasion and metastasis that can be facilitated by the impairment of ECM. It is expected that collagen assembly will be affected even before HIF activation because the *K*_m_ for ascorbate of collagen hydroxylase is twice as high as the *K*_m_ for HIF hydroxylases. Nothing, however, is known about the effect of Ni(II) on collagen hydroxylation and assembly.

The destruction of ECM selects for cells that can proliferate without attachment, which can be tested as the loss of anchorage dependence. Indeed, the loss of anchorage dependence is well documented in human and hamster nickel-transformed cells (Biedermann and Landolph 1987; Conway and Costa 1989; Costa et al. 1979; Lin and Costa 1994). Almost all nickel-transformed clones are also capable of producing tumors in nude mice (Costa et al. 1979). Recently, we have shown that the expression of HIF-1α is required for the induction of soft agar growth by Ni(II) (Salnikow et al. 2003). Under the concept of our hypothesis, it is conceivable that the disruption of ECM and activation of hypoxia-like stress inside cells will provide perfect conditions for selection of cells with neoplastic phenotype. Additionally, inactivation of DNA dioxygenases may introduce mutations preserving selected phenotypes. Nickel is a weak mutagen; therefore, one assumption would be that the *K*_m_ for ascorbate for these enzymes is rather low. Alternatively, DNA dioxygenases repair N1 alkylated adenines, which are not major mutagenic lesions.

If the hypothesis discussed here is true, one may predict that supplementation with ascorbate should alleviate carcinogenic effects of Ni(II). Although the direct data are not available and further investigations are needed to demonstrate protective effect of ascorbate in Ni(II) carcinogenesis, many data demonstrate preventive effects of ascorbate supplementation against Ni(II) toxicity: prevention of oral Ni(II) toxicity in weanling rats (Chatterjee et al. 1979); suppression of nickel-induced lipid peroxidation in human platelets and placenta (Chen and Lin 1998, 2001) and in mouse and rat liver (Chen et al. 1998a, 1998b; Das et al. 2001); decrease in nickel-induced cytokine production and elevated serum levels of hepatic transaminases in rats (Chen et al. 1998a); reversion of nickel-caused decrease in hepatic glutathione level and superoxide dismutase, catalase, and glutathione peroxidase activities in rats (Das et al. 2001); alleviation of clastogenic effects of Ni(II) in murine bone marrow (Dhir et al. 1991); and prevention of DNA damage and increase of viability of human lymphocytes cultured with Ni(II) (Osipova et al. 1998). Osipova et al. (1998) proposed using ascorbate as an antimutagenic agent on nickel-exposed workers. It is noteworthy that in contrast to reports of aggravation by ascorbate of nickel-mediated *in vitro* toxicity (Kasprzak and Hernandez 1989), consistent with the known generation of reactive oxygen species in metal–ascorbate solutions (Buettner and Jurkiewicz 1996), no such effects have been thus far observed in nickel-exposed and ascorbate-supplemented cells and tissues.

## Conclusion

Recent investigations clearly show that the cellular ascorbate is greatly depleted by exposure to Ni(II) compounds, which results in the inhibition of prolyl hydroxylase activity and activation of HIF-1α and expression of hypoxia-inducible genes. The addition of ascorbate reverses these as well as a variety of other toxic effects of Ni(II) in cultured cells and animals. All this indicates that ascorbate may be the major target in nickel-induced toxicity and carcinogenesis. However, the hypothesis of the key role of ascorbate in Ni(II) carcinogenesis awaits further testing because the critical experiments are yet to be performed. Among them, most important is testing nickel carcinogenicity in mice or rats with mutations in l-gulono-γ -lactone oxidase that make them unable to synthesize ascorbate. Monitoring levels of ascorbate during cell transformation *in vitro* or during tumorigenesis *in vivo* is another important task. The elucidation of the role of ascorbate depletion in the development of nickel-induced neoplasms could serve as a basis for prevention and treatment of Ni(II) and, perhaps, also other metal-induced human cancer. Bearing this in mind, we quote Cameron et al. (1979), from a historical paper published 25 years ago: “We agree and believe it to be essential that extensive studies of ascorbic acid in cancer be made without delay.”
